# Lower back pain in physically demanding college academic programs: a questionnaire based study

**DOI:** 10.1186/1471-2474-8-67

**Published:** 2007-07-13

**Authors:** Graham Brennan, Amir Shafat, Ciarán Mac Donncha, Carmel Vekins

**Affiliations:** 1 Department of Physical Education and Sports Sciences, University of Limerick, Limerick, Ireland

## Abstract

**Background:**

Lower back pain (LBP) is ranked first as a cause of disability and inability to work, and is expected to affect up to 90% of the worlds population at some point in their lifetime. The annual first time incidence of LBP is 5%, and the annual prevalence (*i.e*. those suffering at time of questioning) is between 15 and 63%. Prospective studies demonstrate that low back problems do not display a six-week spontaneous recovery pattern, as was once believed. The condition is regularly seen to worsen over time, becoming a chronic disorder, influenced by both physical and psychosocial factors.

**Methods:**

The current study assessed the level of LBP amongst students engaged in educational programs that were physically demanding, and its influence on lower back problems. A 1-year retrospective questionnaire consisting of 37 closed, open and multi-choice questions was designed to ascertain self-reported information on the occurrence, cause and type of LBP. Treatment, care seeking and general knowledge regarding LBP were also recorded. Students were enrolled in BSc Equine Science, BSc Physical Education and BSc Sports & Exercise Science degree programs and a total number of 188 valid questionnaires were collected.

**Results:**

The self reported, anthropometrical data for participants in this study are: age 20.9 ± 2.7 yrs; height 171.8 ± 9.3 cm; weight 66.7 ± 10.4 kg; female 64% (n = 120), male 36% (n = 68). The overall self reported prevalence of LBP was 32% (n = 61). Within the LBP population, 77% reported their problem as recurring. Two factors showed significance as having an influence on LBP. They were age (21.6 ± 3.5 yrs, p = 0.005) and hours of personal training physical activity (14.0 ± 8.2 hrs per week, p = 0.02). LBP sufferers also displayed poor management of their condition and an interest in education and treatment of their problem.

**Conclusion:**

The current study revealed high prevalence of LBP consistent with that of the literature, and unveiled a recurrence rate and behavioral habits of sufferers, which are warning signs of a more chronic state to come. Novel data presented here offers strong support for the need for prospective injury tracking, plus educational intervention and treatment aimed at prevention of LBP.

## Background

Lower back pain is ranked first as a cause of disability and inability to work, and expected to affect up to 90% of the worlds population at some point in their lives [1]. Lower back pain is a complex condition, influenced by a number of factors and often a challenge when trying to identify any singular cause or even a single major factor [2]. The annual first time incidence of lower back pain is 5% [1], and the annual prevalence between 15 and 63% (*i.e*. those suffering at time of questioning) [3,4]. Prospective studies demonstrate that low back problems do not display a six-week spontaneous recovery pattern [5], as was once believed. The condition is regularly seen to worsen over time, becoming a chronic disorder, influenced by both physical and psychosocial factors [6,7].

Cross-sectional data demonstrate that initial onset of lower back pain is expected to occur around the mean age of 30 [8], and peaking in occurrence between the ages of 45 and 60 years [1,8]. However, lower back pain is common in both older and younger adults. Nyland and Grimmer (2003) stated that an early emergence of lower back pain and the increase duration of suffering may go so far as to decrease performance of duties in any physically active vocation, in their case, physiotherapy. Early onset of lower back pain may be even more important in vocational training where physical activity is part of the curriculum and profession such as physical education teaching [9].

The influence of physical activity in relation to lower back pain has been observed as associative [4,10], non-associative [11] and even protective [8,12]. Jacob and colleagues [11] identified the specific factors of high occupational activity (lifting and loading) and low perception of general health as contributory in a general population. Hestbaek *et al*. noted how the so-called protective effect of a sedentary occupation was lost once physical activity is undertaken [13]. But still no firm associations between physical activity and lower back pain have been made. It is proposed by Adams (2002) [14], that a 'U shaped' curve best describes the correlation between lower back pain and physical activity. Sedentary lifestyles, as well as extremely active lifestyles are associated with increased prevalence while moderate activity seems protective [15]. It is not implied that inactivity causes lower back pain, nor that high activity is a result of lower back pain, but studies have indicated that engaging in higher intensities of physical activity, particularly with a history of lower back pain is associated with lower back pain [9,13,16], or extremity of flexion and/or load in the lumbar region [17,18]. In contrast, once lower back pain has occurred, low activity levels have been associated with prolonging suffering and lengthening time to recovery [8,11,12,19].

Rehabilitation and care seeking habits of the sufferer can be poor and often affected by psychosocial factors [20], financial compensation [3,21] and even a simple lack of knowledge. Educational intervention and reducing waiting time before primary examination has resulted in a reduction in financial compensation claims both in Australia [20] and Ireland [21,22]. This indicates a possible light at the end of the tunnel and that lower back pain can be controlled and the burden lightened physically and financially.

Lower back pain is a heterogeneous condition, which may contribute to variation in reported prevalence. In the absence of 'a gold standard to evaluate low back pain' [23] questionnaires are considered reliable measurement tools [24] for the assessment of this condition.

The current study assessed the level of lower back pain amongst students engaged in educational programs that included a physical activity component, which constituted a quarter of their academic program. The primary objective of the study was to examine the impact of physically demanding academic programs on the occurrence of lower back pain. Additional objectives were to gather baseline statistical data on the current prevalence, state of recovery from the condition (and any other injuries reported), possible causes and type of lower back pain and educational awareness in regards to lower back pain.

## Methods

### Definition of lower back pain

Lower back pain was defined in this study as an episode of pain or discomfort that interrupted normal daily activities and/or required treatment or consultation in the lower back. Lower back was defined as the region between the twelfth rib and the gluteal folds on the posterior aspect of the trunk [7].

### Apparatus

A 1-year retrospective questionnaire consisting of 37 closed, open and multi-choice questions was designed to ascertain self-reported information on the occurrence, cause and type of lower back pain and levels of physical activity. The following question was asked to ascertain lower back pain: 'Do you now, or have you between September 2002 and November 2003 suffered from any lower back problems'. Treatment, care seeking and general knowledge regarding lower back pain were also recorded. Further, information relating to recurrence, present status of the lower back problem, coping strategies, medical assistance and rehabilitation was also collected. Participants who reported lower back pain were asked further questions regarding cause, location, absenteeism and influence on daily function. A diagram was used to help identify and mark the exact location of the lower back pain. Two open questions were asked: 'What type of activity were you doing?' and 'For your most recent back problem, what did you feel was the actual movement or mechanism that caused the problem?' Finally, the present and desired level of knowledge regarding lower back pain was examined with the questions; 'Do you feel enough back education is received in your program of study?' and 'Would you be interested in attending a back care program?'

### Participant recruitment

After approval by the University of Limerick Research Ethics Committee, questionnaires were administered during class time, thus ensuring high questionnaire return.

A random sample of students participated in this study; students were enrolled in BSc Equine Science, BSc Physical Education and BSc Sports & Exercise Science degree programs at the University of Limerick. These academic programs were chosen because a physical activity element represents approximately a quarter of their program of study, and a major interest of this study was the influence of physically demanding college academic programs on lower back pain. Due to the large number of students involved, a fraction of each year was surveyed. This study collected data from at least thirty percent of the students from each academic program, and where total class size was small more students were included (see Table 1). Forty-three questionnaires were collected from a total of 67 students in BSc Equine Science, 70 from a total of 236 BSc Physical Education, and 75 from a total of 159 BSc Sports and Exercise Science students. A total of one hundred and ninety questionnaires were administered between November 2003 and January 2004. One hundred and eighty eight valid questionnaires were returned representing a response rate of 99%. The questionnaire investigated physical activity habits and injury status over the previous 12-month period.

**Table 1:** Total class sizes per year, per academic program at time of investigation and percentage of students participating in the study.

**Course**	**Students participating in the study per year of study (% of total)**				**Total**
		
	**First**	**Second**	**Third**	**Fourth**	
Equine Science (n = 43)	7/23 (30%)	9/20 (45%)	15/15 (100%)	12/12 (100%)	43/67 (64%)
Physical Education (n = 70)	37/80 (46%)	16/71 (22%)	12/42 (29%)	5/43 (12%)	70/236 (30%)
Sports and Exercise Science (n = 75)	10/40 (25%)	33/47 (70%)	14/37 (38%)	18/35 (51%)	75/159 (47%)
Total (n = 188)	54/143 (38%)	138 (42%)	41/92 (45%)	35/89 (39%)	188/462 (41%)

### Analysis

All data were analyzed using SPSS version 12.0 (Chicago, Illinois, USA). Statistical significance was set at p ≤ 0.05. Skewness of the data was used and as indication of normality. The skewness of the quantitative data (height, weight, age, hours of activity and number of sports participated in) was calculated by dividing the raw skewness value by the standard error for skewness. The resultant value was interpreted as a Z score, (i.e., values ± 1.96 exceed p ≤ 0.05, and values ± 2.58 exceed p ≤ 0.01). Data were considered normally distributed if the Z value did not exceed ± 2.0 [25]. In the case of non-normal data non-parametric analysis was used. All data, with the exception of height were found to be non-normal. In the case of normally distributed data, independent sample *t*-tests were used to examine differences between individuals reporting with and without low back pain. Mann-Whitney U rank tests were used to examine differences between individuals with and without low back pain on the following variables: age, weight, body mass index, hours of academic program physical activity, hours of personal training physical activity, number of sports participated in within academic program and the number of sports participated in within personal training. A Pearson's chi-square test was used to examine the association of back pain occurrence with gender and academic program taken. The assumptions for chi-square were met for this comparison. All data are presented as Mean ± SD. The following equation ES = (M_1 _- M_2_·s^-1^) was used to determine effect size; where for example M_1 _= the mean age of participants without lower back pain and M_2 _= the mean age of non-lower back pain participants and s^-1 ^= the pooled standard deviation [26].

## Results

### Participant characteristics

The self reported and anthropometrical data for participants in this study are: age 20.9 ± 2.7 yrs; height 171.8 ± 9.3 cm; weight 66.7 ± 10.4 kg; body mass index 22.6 ± 2.7 kgm^-2^; female 64% (n = 120), male 36% (n = 68).

### Prevalence

The overall self reported 12-month prevalence of lower back pain was 32% (n = 61). Within the lower back pain population, 77% reported their problem as recurring. Surprisingly, 14% of questionnaires contained an unsolicited comment describing their lower back pain problem as constant or ongoing. When compared to all other injuries by type and site, lower back pain was more frequent than sprain at 24% and ankle injuries at 15% respectively.

### The factors associated with low back pain

Two factors showed significance as having an association with lower back pain. They were age and hours of personal training physical activity. All other factors investigated (height, weight, body mass index, gender, academic program, year of study, hours of academic program physical activity, number of sports participated in within academic program and the number of sports participated in within personal training) were not found to be significant (see Table 2). The mean age of those reporting lower back pain was 21.6 ± 3.5 yrs vs. 20.6 ± 2.1 yrs for non-lower back pain (p = 0.005, see Table 2). The hours of personal training physical activity was 14.0 ± 8.2 hrs per week for lower back pain and 11.2 ± 7.5 hrs per week for non-lower back pain (p = 0.02, see Table 2). Also, students reported hours of academic program physical activity ranging between 8.0 ± 3.1 to 8.4 ± 6.3 hours per week. In answer to the question "how many different sports do you take part in?" responses ranged from 6.8 ± 4.9 to 8.4 ± 7.3 separate sports as part of their academic curriculum and 4.0 ± 2.7 to 4.4 ± 2.3 separate sports as part of their personal training. These factors were similar in lower back pain and non-lower back pain groups.

**Table 2:** Measured characteristics of participants with and without lower back pain.

	LBP (n = 61)	Non-LBP (n = 127)	Significance	Effect Size
Age	21.6 ± 3.5	20.6 ± 2.1	p = 0.005	0.380
Height	172.4 ± 8.1	171.5 ± 9.9	p = 0.626	0.009
Weight	66.9 ± 10.2	66.7 ± 10.5	p = 0.802	0.002
Body mass index	22.5 ± 2.9	22.6 ± 2.5	p = 0.772	0.045
Academic sports participated in	8.4 ± 7.3	6.8 ± 4.9	p = 0.833	0.270
Personal Training Sports participated in	4.0 ± 2.8	4.5 ± 2.3	p = 0.068	0.200
Academic hours of physical activity	8.5 ± 6.3	8.1 ± 3.1	p = 0.918	0.097
Personal Training hours of physical activity.	14.0 ± 2.8	11.2 ± 7.5	p = 0.020	0.440

LBP = Lower back pain

### Activity at time of occurrence and mechanism responsible

Self reported responses to questions regarding the cause of their lower back pain, 25% named a team sport as the activity undertaken when the problem occurred, while 24% named lifting as the contributory action. Other activities included; individual sports (20%), horse riding (14%) and lifting (10%). Other activities reported included contact within sports (16%), strength and fitness training (15%) and falls (10%).

### Location and type

With use of the diagram, 39% reported lower back pain in L4–L5, 18% in L1–L3, and 11% reported 'low back/non-specific'. All other regions (sacro-iliac joint, sacrum, coccyx, lumbo-sacral joint, posterior superior iliac spine) ranged between 3 and 9% each. When asked to report what type of back condition they suffered from, either diagnosed or self-assessed, participants gave less definitive answers. Twenty three percent replied 'general back pain', while 'back strain' and 'slipped disc' were equally frequent at 10% each. Other conditions included sharp radiating pain, compressed vertebrae, sciatica, osteoarthritis, back strain, muscle spasm, bruising and fracture of the coccyx, and were reported between 1 and 8% each. Non-responders accounted for the remaining percentage.

### Impact and care seeking habits

When asked, "Which health care practitioner did you consult?" physiotherapists were the most frequently attended professionals (26%), general practitioner (13%), chiropractor (8%) and 'other' (10%). Interestingly, 43% reported attending no medical professional for their condition. Thirty-six percent of lower back pain sufferers lost between 1 and 31 days due to their condition, while 5% reported more than 6 months absence from physical activity (work, sport, education). 31% lost less than a day due to lower back pain. Non-responders accounted for the remaining percentage.

Only 36% reported the use of coping strategies. The most commonly reported were lower back and core exercises (10%), stretching (5%), whilst 3% reported 'avoidance' as a coping strategy. Ranging between 1% and 5 %, other strategies included correct warm-up, postural corrections, warm baths, massage and rest. A further, seven percent reported taking prescription medication.

Of those with lower back pain, 48% reported the condition to be healed but recurrent, 32% were receiving treatment but still active in sport, 8% were healed and non-recurrent, 3% were temporarily withdrawn from physical activity and the remainder did not respond to the question. Looking more specifically at the restrictions imposed by lower back pain, 38% reported a reduction in mobility, 36% reported discomfort from sitting or standing for prolonged periods and 15% reported difficulty lifting. The remainder did not respond to the question.

### Education assessment

When asked if enough information was provided within their program regarding lower back pain, 65% of those suffering from lower back pain answered no. Further, 64% were interested in attending a back clinic.

## Discussion

The current study found a 12-month prevalence of 32% and is in general agreement with prevalence rates reported in the literature, which vary from 15% to 63% [3,4]. Nyland and Grimmer (2003)[4] assessed undergraduate physiotherapy students using the Nordic back care questionnaire, and their thorough assessment of lower back pain retrospectively, allowed the investigators to display separately 12 month, one month, one week prevalence. Retrospective studies can produce detailed results, but the prospective approach can further assess change over time and enhance investigations. Palmer *et al*. [27] prospectively revealed 36% prevalence in the general population, which increased to 49% over ten years. The deterioration of lower back pain into a chronic state condition can be examined using a prospective approach, particularly when investigating healing times and impact on the sufferer. This calls into play the usefulness of 'recurrence rates' as a beneficial measurement of the overall impact of lower back pain.

Students in the current study engaged in physical activity, reported a 77% recurrence rate, in agreement with recent literature, which ranges between 60% to 76% [19,28,29]. Recurrence and healing are controversial topics as it has been stated that 90% of lower back pain will resolve itself within one month [1]. However, Croft and colleagues' (1998)[5] prospective work on healing and care seeking showed that although 59% of sufferers did not consult again within six months of injury, only 25% had fully recovered within 12 months. In summary, lower back pain does not spontaneously heal or subside within one month, but persists over a twelve-month period, and the only decline may be in return visits to medical practitioners.

When asked when the low back pain first occurred, some students made a surprising and unsolicited comment. That was, their lower back pain was 'constant' or 'continuous'. This conservatively represented 14% of sufferers and may indicate a chronic state of lower back pain. Chronic lower back pain, seen as a stage in the progressive development from acute to sub-acute and finally chronic, as a result of recurrence [14] has reported rates of 20–30% across a broad range of populations [7,30]. Data presented in the current study may under-represent the actual rate. The mechanisms of change and deterioration to chronic state may be as difficult to assess retrospectively as the initial cause of lower back pain [2]. However, physically active students in the current study did display behaviors that may be of concern in relation to the development of chronic lower back pain. These include: 39% never attended a medical practitioner; few reported using a coping strategy ('core body exercises' highest at only 10%), a small but important 6% reported using prescribed medication and 3% stated 'avoidance' as a coping strategy. Together, these observations indicate a tendency towards passive coping. Passive coping is strongly associated with disabling neck and back pain [31] and thoroughly contrary to findings that active rehabilitation (physical or cognitive) is superior to passive coping [32]. In light of these findings the sufferers in the current population are likely to be following the path of lower back pain sufferers observed by Croft *et al*. [5] and Mercado *et al*. [31]. They may find themselves suffering longer periods of absenteeism and reduced daily functionality. The high prevalence (32%) and high recurrence (77%) suggest that lower back pain in this population is a serious health concern.

Increasing this concern may be the fact that, similar to Nyland and Grimmer [4], the population sampled are undergraduate students, with an average age approximately a decade younger than those observed in the majority of studies on lower back pain.

The literature typically reports the onset of lower back pain at approximately 30 years of age [12] and peaking in prevalence within middle age [8]. Lower back pain sufferers in this study, however, were younger at 21.6 years. But lower back pain is not uncommon in younger populations, and has been observed from early teens and onwards [33]. Rates vary throughout adolescence as Prendeville and Dockrell [34] found a high lifetime prevalence of 42%, in 13 to 17 year olds, while Nyland and Grimmer's [4] reported ages 20 and 21 years. Further, their risk of lower back pain increased after year one of physiotherapy training. These were final year students, indicating an effect of long-term (3 to 4 year college career) exposure to stress and strain on the lower back. No association to year of study was observed in the current population, but the average age of 21.6 years for lower back pain sufferers, does coincide with Nyland and Grimmer's [4] final year students (21.4 yrs). The onset of lower back pain at this low age brings forward the expected emergence of lower back pain by approximately 9 years. This increase in time exposed to risk factors may also increase wear and tear on the lower back and therefore the risk of injury.

The current study reports high prevalence and highly significant recurrence rates for lower back pain. As reported by Nyland and Grimmer [4], entering the workforce with poor lower back health is not uncommon amongst students following a similarly active academic program. Stergioulas *et al*. [9] reported 63% prevalence amongst physical education teachers, attributing 'no personal training' and occupational factors such as 'lifting gym instruments' and 'helping students into flexing posture' as significant risk factors. The high prevalence of lower back pain in a young and active population is a worrying trend, especially in light of similarities in age to other lower back pain studies on active populations. The physical activity type associated with lower back pain warrants further investigation and the logical progression for the current study is an examination of physical activity in a number of different forms, to establish links between lower back pain and activity.

Findings in the literature vary regarding the impact of activity and exercise, but studies on elite athletes and sports involving hyper-flexion and extension have reported higher prevalence of lower back pain [10]. Links have also been found between occupational activities (lifting and loading) and lower back pain [11,13]. Adams *et al*. [14] proposed a U-shaped association with lower back pain. A simple association between low activity levels and lower back pain may be an inappropriate claim, as lower back pain may be more an effect than a cause of sedentary lifestyle. Athletes, however, have suffered lower back pain due to long duration of training [9,13,16], or extremity of flexion and/or load in the lumbar region [17]. In the current study, 'hours of physical activity in personal training' was the only factor significantly associated with lower back pain, and those reporting lower back pain carried out an average of 14.0 ± 8.2 hrs per week, while non-sufferers completed 11.2 ± 7.5 hrs per week of personal training.

Key significance is the fact that personal training is one of a number of activity types participated in by the students. Initially considerations included investigating if a threshold for lower back pain exists somewhere between 11 and 14 hours of personal training. But lower back pain is a multi-factorial condition [35], and can be initiated by combined influence of genetics, environment and exposure to risk factors. The conclusion therefore is that we may simply be observing a culmination of factors with perhaps several factors combining to cause lower back pain. Details regarding other physical activity in the current study are as follows: students reported hours of academic program physical activity ranging between 8.0 ± 3.1 to 8.4 ± 6.3 hours per week, in answer to the question "how many different sports do you take part in?" responses ranged from 6.8 ± 4.9 to 8.4 ± 7.3 separate sports as part of their academic curriculum and 4.0 ± 2.7 to 4.4 ± 2.3 separate sports as part of their personal training. This may be contributing to an excessive strain on the lower back. We are reluctant to accept a reduction in personal training hours as a way to alleviate lower back health, because of other health implications of such interpretation, the self reported nature of current data, and the need for further clarification of the physical activity in this population.

As stated, the current study was retrospective, but Verni *et al*.'s [16] prospective work with fin swimmers, enabled the authors to identify the peak occurrences for lower back pain through injury tracking. This established the time of year most associated with lower back pain and concluded that poor fitness and technique early in the season, and exhaustion late in the season were contributory factors. This further supports both a prospective approach and a U-shaped association with activity, and indicates a multi-factorial assessment of cause in lower back pain.

A number of recent studies have investigated physiotherapy versus education intervention, and shown that education, even alone, may aid the sufferer as much as physiotherapy. Alston and O'Sullivan [21], Frost *et al*. [36] and Uderman *et al*. [37] all showed education intervention was as, and even more effective than physiotherapy alone in improving pain management and pain resolution. When undergraduates in the current study were asked if they 'received enough information regarding lower back pain on their academic program of study', a majority expressed the opinion that not enough lower back pain information was provided. Equally, interest in a back education program was strongly expressed as 'yes' when asked if they would attend a back clinic. The information requested included 'proper lifting techniques' and 'background information on prevention of lower back pain'.

This indicates an interest in lower back pain management and back care, suggesting that the avoidance habits observed at present may not be by choice, but in-fact due to lack of choice or awareness of constructive pathways to recovery. Population based intervention programs [38,39] have provided empirical evidence that provision of mass media based education, and the use of standardized instruments can improve coping, recovery and quality of life for the lower back pain sufferer in a quantitatively verifiable way. The current study indicates that a young skilled and educated population, is emerging as poor in low back health state, poor in management behavior, and yet eager to become more informed and move toward self-management of the problem if given the opportunity. It is unclear whether that opportunity is at present available to them. We propose that all student populations be taught good back health practices.

## Conclusion

The current study revealed high prevalence of lower back pain consistent with that of the literature, and unveiled a recurrence rate and behavioral habits of sufferers, which are warning signs of a more chronic state to come. In this study, the condition was more prevalent than all other injuries, and had two main factors associated with it, age and hours of personal training physical activity. Associative factors vary across studies. Age as a significant factor is of great concern in this young population entering the workforce and en route to a greater exposure to risk factors. Personal training may only be the tip of the iceberg as regards the accumulation of stress and strain on the lower back. Without quantifiable and structured assessment, tracking and educational intervention, this condition may not resolve itself, but become more prevalent and indeed more chronic amongst sufferers. Novel data presented here offers strong support for the need for prospective injury tracking, plus educational intervention and treatment aimed at prevention of lower back pain.

### Limitations

Limitations in this study restrict our data to self-reported information. Also, the study is not prospective in nature. The study population does not reflect a more 'general' population due to the limited mean age and narrow age range, and the study population is a group of university students rather than a 'normal' population.

## Competing interests

The author(s) declare that they have no competing interests.

## Authors' contributions

CV and GB designed the study. AS, CMD and GB analyzed and interpreted the data. All authors read and approved the final manuscript.

## Pre-publication history

The pre-publication history for this paper can be accessed here:


